# Multicomponent High-throughput Drug Screening via Inkjet Printing to Verify the Effect of Immunosuppressive Drugs on Immune T Lymphocytes

**DOI:** 10.1038/s41598-017-06690-2

**Published:** 2017-07-24

**Authors:** Moonhyun Choi, Jangsun Hwang, Jonghoon Choi, Jinkee Hong

**Affiliations:** 1 School of Chemical Engineering and Material Science, Chung-Ang University, 84 Heukseok-ro, Dongjak-gu, Seoul, 06974 Republic of Korea; 2 School of Integrative Engineering, Chung-Ang University, Seoul, 06974 Republic of Korea

## Abstract

High-throughput drug screening based on a multi-component array can be used to identify a variety of interaction between cells and drugs for suitable purposes. The signaling of immune cells is affected by specific proteins, diverse drug combinations, and certain immunosuppressive drugs. The effect of a drug on an organism is usually complex and involves interactions at multiple levels. Herein, we developed a multilayer fabricating system through the high-throughput assembly of nanofilms with inkjet printing to investigate the effects of immunosuppressive drugs. Immunosuppressive drugs or agents occasionally cause side effects depending on drug combinations or a patient’s condition. By incorporating various drug combinations for understanding interaction between drugs and immune cells, we were able to develop an immunological drug screening kit with immunosuppressive drugs. Moreover, the ability to control the combination of drugs, as well as their potential for high-throughput preparation should be of great benefit to the biomedical and bioanalytical field.

## Introduction

Immunosuppressive drugs are important for end-stage patients with organ diseases who require an organ transplant to live. These drugs suppress the activation of immune cells, prevent acute organ rejection during surgery, and play a significant role in increasing the survival rate of patients who received organ transplants, by more than 80% after 1 year^1^. Immunosuppressive effects are exerted through various mechanisms. In the case of immunosuppressive drugs used in this study, rapamycin (RAPA) inhibits interleukin (IL)-2 and other cytokine receptor-dependent signal transduction mechanisms, via action on mechanistic target of rapamycin (mTOR), and blocks the activation of T and B lymphocytes^2, 3^. Cyclosporine A (Cyc A) can be combined with the cytosolic protein cyclophilin^4^. Mycophenolic acid (MPA) inhibits T and B lymphocyte proliferation by inhibiting inosine monophosphate dehydrogenase, which is involved in the biosynthesis of guanine nucleotides that are required for the development of lymphocytes^1^.

However, the effect of these drugs in an organism is complex and involves interactions at multiple levels^5^. Immunosuppressive drugs or agents occasionally cause side effects depending on drug combinations or the patient’s condition. In addition, most immunosuppressive drugs are small molecules, indicating that they have features characteristic of small molecules^6, 7^. The handling and application of these molecules are associated with certain obstacles such as low therapeutic effectiveness^8^, structural instability under physiological conditions^9, 10^, short half-lives, highly controlled release kinetics^11^, or small loading amounts^12^. These challenges lead to short release time scales, limited therapeutic scope, ill-defined release mechanisms, and structural destabilization, which have greatly hindered the utility of functional molecules.

To effectively deliver immunosuppressive drugs, significant research has been conducted using traditional injection needles^13^, chitosan nanoparticles^14^, and nanogels^15^. Almost all studies have focused on delivering the optimal dose of an immunosuppressive drug with minimal side effects^16^. Although there are various delivery methods, currently it is difficult to predict whether adverse reactions will occur in patients owing to the potentially enormous amount of drug candidates and combinations, and also because drug effects can differ depending on a patient’s gender, age, weight, condition, among others. Combining immunosuppressive drugs can result in a similar immunosuppressive potency whilst lowering the side effects of the drugs, compared to their individual use^17^. Therefore, it is imperative to introduce methods of analyses, which could determine the positive or negative effects of immunosuppressive drug combinations, with respect to a patient’s immune system.

High-throughput screening allows for fast and effective individual diagnoses. A technical analysis of a biological specimen is used to determine the presence or absence of specified parent drugs or their metabolites, which allows researchers to quickly conduct millions of chemical, genetic, or pharmacological tests^18^. Through this process, one can rapidly identify active compounds, antibodies, or genes that modulate a particular biomolecular pathway. In particular, immunosuppressive drug screening is essential to determine the subtle differences among individuals.

Through the incorporation of immunosuppressive drugs with coated nanofilms, the previously mentioned obstacles and challenges can be solved. Coating nanofilms involves developing a thin film of a material by bridging the physical, chemical, or biological characteristics of building molecules, which can facilitate the handling of a material for a desired purpose^19^. The inkjet-assisted multilayer assembly system, an effective method proposed to develop multilayered nanofilms, is driven by molecular interactions and allows excellent modification of a surface^20–24^. Inkjet printing method is very useful for applying expensive biomolecules, such as drugs, proteins, DNA, growth factors, and so on, because activity loss of highly sensitive materials can be minimized by reducing direct contact between substrate and materials solution. Furthermore, surface coating can be formed on fairly large area in small quantity. The inkjet-assisted technology is widely used to fabricate ultrathin coatings and complex materials from synthetic and natural polymers, nanoparticles, and fibers with a variety of functionalities. Further, this process can control the thickness, permeability, strength, porosity, and environmentally responsive properties.

**Figure 1:**
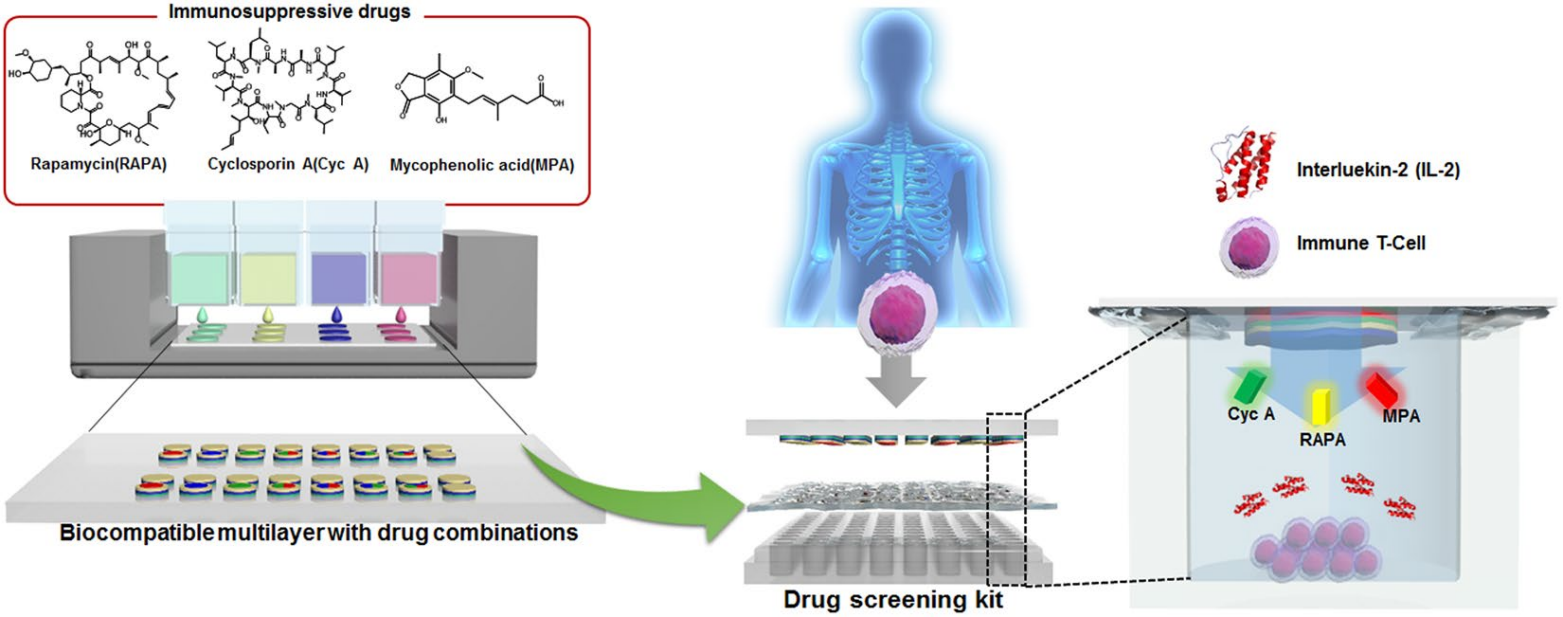
Schematic illustration of a three layered drug screening kit to effectively analyze various effects of medication for immunosuppression with inkjet printed biocompatible nanofilms including various drug combinations (The figure including inkjet printing image, Human torso image, the Immune T-Cell and the drug screen kit was drawn by M.C).

Herein, we investigated how immune T cells are affected when three immunosuppressive drugs (RAPA, Cyc A, and MPA) are used in combination or as individual treatments. To control stability and release pattern, we fabricated poly-L-lysine (PLL)/hyaluronic acid (HA) multilayered nanofilms that incorporated immunosuppressive drugs with mainly electrostatic interaction and partially hydrogen bonding. Additionally, for ease of practical use, we prepared a three-stage kit with the inkjet-assisted multilayer assembly technique for rapid screening *in vitro* (Fig. 1). The ready-made kit would help doctor or medical team rapidly anticipate side effects from medicines depending on patients.

## Results

**Figure 2:**
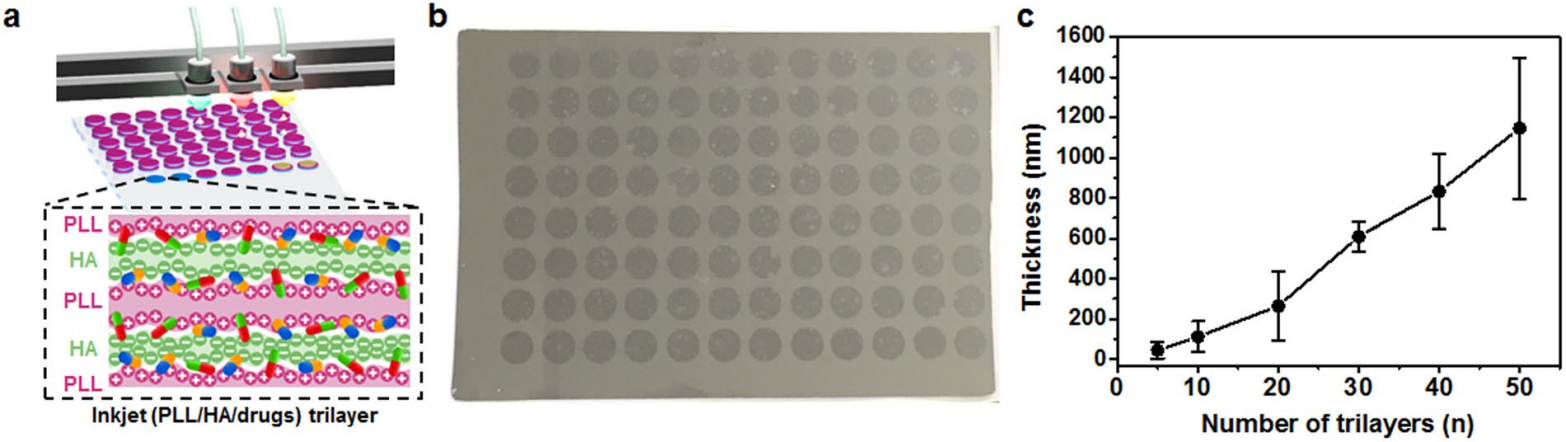
(**a**) Schematic illustration of fabrication of printed (PLL/HA/drugs) trilayer based mutilayer nanofilms for the high-throughput immunosuppressive drug screening and chemical structures of immunosuppressive drugs (MPA, RAPA, Cyc A) (The figure was drawn by M.C). (**b**) Digital image of a 96-well plate as a printed shaped on the silicon wafer. (**c**) Thickness profile analysis of (PLL/HA)_*n*_, _*(n = bilayers)*_ printed multilayer nanofilms as measured by a mechanical profilometer.

Inkjet-based multilayer assembly facilitates the generation of a variety of characteristics (loading amount, thickness, shape, components, among others) on the multiple independent deposition spots. Inkjet printing was conducted using different hydrophilic substrates (glass and silicon wafer). The inkjet printer consisted of a 6-ink reservoir that can store a material solution. Unlike heat or bubble printing techniques, a piezoelectric printing system can fabricate a 1.5–2 pL droplet without damaging the materials and easily deposit the droplet onto desirable locations using a software program (Photoshop CS6). To fabricate independent ink droplets containing functional materials, we filled PLL, HA, and immunosuppressive drugs into each ink reservoir, which can be easily controlled by the software program. By alternating the droplets of materials, we successfully prepared a high-throughput assembly with a patterned multilayer nanofilm. Fig. 2a illustrates this excellent high-throughput assembly. We prepared printed multilayer nanofilms, shaped as a 96-well plate (Fig. 2b); these nanofilms were made of various combinations of immunosuppressive drugs on the single substrate.

To handle immunosuppressive drugs comfortably, we used a mixture of water with DMSO (1:1 v/v) to improve their solubility and efficacy. DMSO is an excellent polar aprotic solvent that dissolves both polar and nonpolar compounds, and is miscible in a wide range of organic solvents as well as water. Accordingly, the DMSO/water co-solvent is important with regard to the ionization of polyelectrolytes and concurrent handling of immunosuppressive drugs.

Additionally, to incorporate the drugs stably and quantitatively, printed multilayer nanofilms with biocompatible PLL and HA were designed for providing a stable environment to immunosuppressive drugs. PLL is a homopolypeptide, is widely used as an attachment factor which improves cell adherence^25^ and in drug delivery^26–29^, and contains a positively charged hydrophilic amino group. HA is a ubiquitous carbohydrate polymer that is part of the extracellular matrix^30^, and non-sulfated glycosaminoglycan distributed widely throughout connective, epithelial, and neural tissues. It is an anionic natural polymer and has been used in various fields, such as cancer therapy^31, 32^, wound healing^33^, cell migration^34^, skin care^35^, cosmetic surgery^36^ and so on. (PLL/HA/drugs) trilayer-based nanofilms, on the glass and silicon wafer, were fabricated mainly by electrostatic interaction and partially by hydrogen bonding. PLL and HA are strongly influenced by electrostatic interaction, and building blocks of nanofilm and immunosuppressive drugs are connected by hydrogen bonding. Printed (PLL/HA)_*n*_ multilayered nanofilms were more bulky than general (PLL/HA)_*n*_ films from dipping method owing to the absence of the washing step, which is an essential step in general dipping process, during the surface adsorption process of materials. Nevertheless, a constant droplet volume enables deposition on the surface, which results in the linear growth of the printed multilayered nanofilms (Fig. 2c). With accurate volume droplet control and the use of the patternable technique without a washing step, we prepared patterned (PLL/HA/drugs)_*n*_ trilayer-based nanofilm arrays that have the potential to be utilized in *in vitro* combinatorial drug screening for high-throughput analysis.

**Figure 3:**
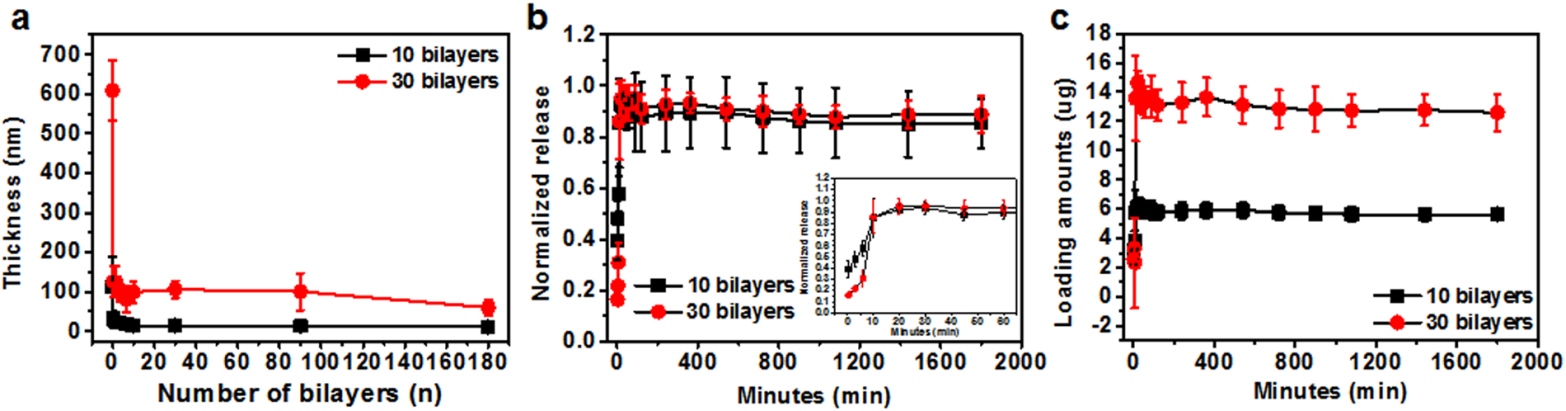
(**a**) Decreasing film thickness of printed (PLL/HA/FITC)_*n(n = number of trilayers)*_ trilayer-based nanofilms over time. (**b**) Normalized and (**c**) total release profiles of printed (PLL/HA/FITC)_*10, 30*_ nanofilms during 30 h.

We can determine regulation of the immune system by evaluating interactions of cells with the released immunosuppressive drugs from the printed nanofilms. Because the interactions between T cells and immunosuppressive drugs should be identified within 12 hours, printed (PLL/HA/drug)_*n*_ trilayer-based nanofilms require rapid degradation for burst release. Printed (PLL/HA/drugs)_*n*_ trilayer-based nanofilms without the washing step would mean that they deposit onto the surface in bulk, compared to conventional LbL methods. Therefore, in cases of the bulk state, water molecules can rapidly penetrate printed (PLL/HA/drugs)_*n*_ trilayer-based nanofilms. Contact of water molecules and film components quickly occurs, which results in pH change inner films, a fast degradation of the films and burst release of molecules in the films. We confirmed the degradation of films and release pattern of molecules in the film by using fluorescein isothiocyanate (FITC) as a release model molecule of immunosuppressive drugs. Properties (molar mass, solubility, among others) of FITC are analogous to those of MPA (Table S1, see the Supporting Information for details). Through the decrement of printed film thickness in the FBS buffer, we can deduce the degradation rate of printed (PLL/HA/FITC)_*10,30*_ trilayer-based nanofilms in 3 hours (Fig. 3a). In as little as few minutes, the thickness of the printed films in the buffer sharply decreased by as much as 80% in both 10 trilayers and 30 trilayers cases.

In addition, FITC molecules in the printed (PLL/HA/FITC)_*10,30*_ nanofilms were explosively released with a fast degradation rate (Fig. 3b). In 10 minutes, the amount of released FITC molecules was more than 85%. The release patterns from different trilayers were similar, but the total amount of released FITC molecules can be controlled by changing the number of layers. This means that printed nanofilms are capable of controlling the amount of released immunosuppressive drug. For printed (PLL/HA/FITC)_*n*_ nanofilms, the total released amount from the 10 and 30 trilayers was 6.22 and 14.66 μg/cm2, respectively (Fig. 3c).

**Figure 4:**
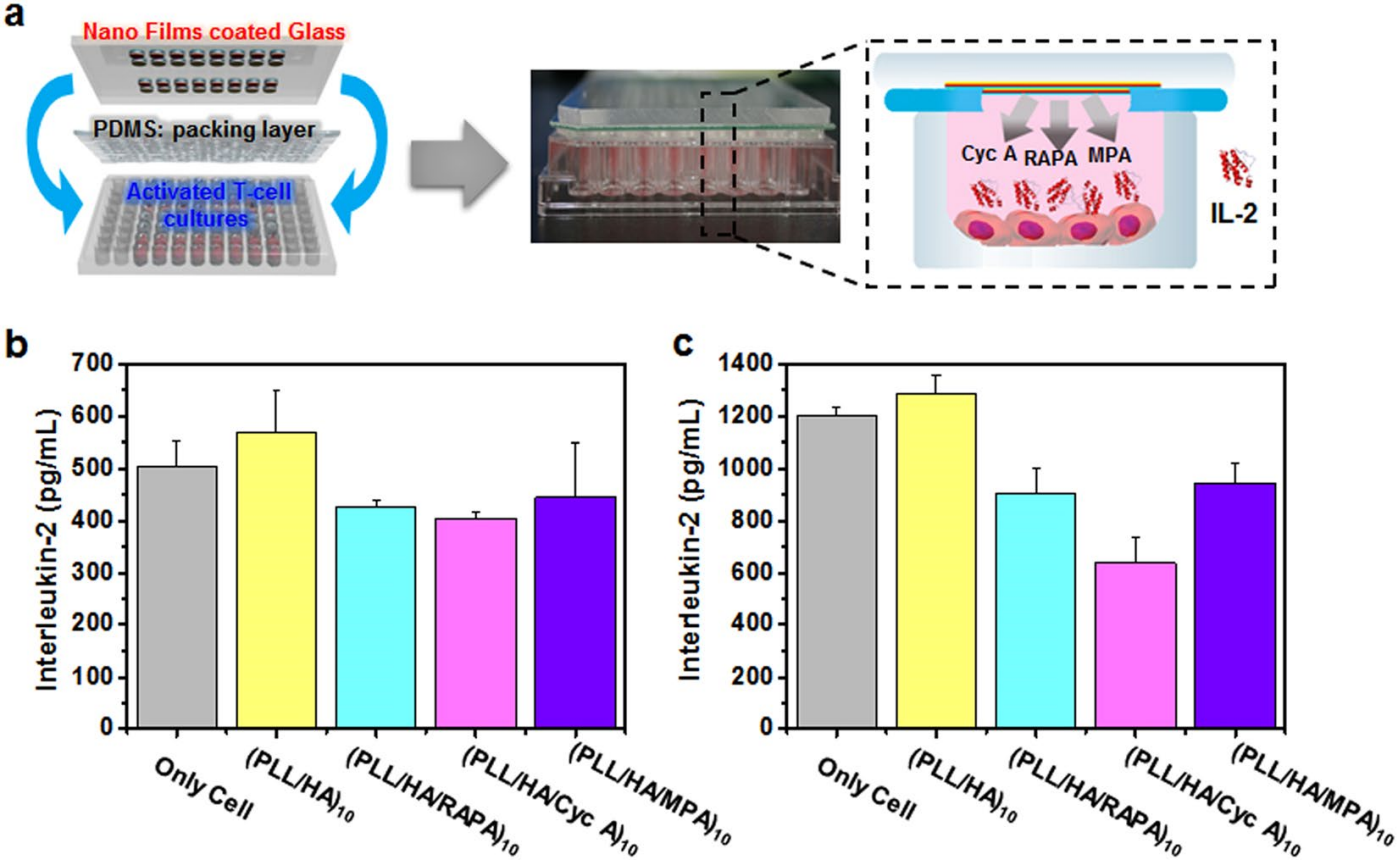
(**a**) Schematic illustration of the high-throughput drug screening process (The figure was drawn by M.C). Phytohaemagglutinin (PHA) treatment (**b**) before and (**c**) after each of the immunosuppressive drug tests for confirmation of the effects of immunosuppressive drugs in the printen nanofilms.

We developed a three-layered kit for a drug screening system by sandwiching three layers: an activated immune T cell culture plate on the bottom, PDMS mold as a packing layer in the middle, and drug-incorporated printed nanofilms on the top (Fig. 4a and Figure S3, see the Supporting Information for details). Using the three-layered kit, Fig. 4a illustrates that IL-2 was released through this system through the interaction between T cells in the wells of the plate and immunosuppressive drugs released from the films. IL-2, an indicator of T cell activation, is produced mainly by activated T lymphocytes, which help with the body’s immune system, cell tolerance, and immunity^37^. As activation of T cells was inhibited by immunosuppressive drugs, the total amount of secreted IL-2 decreased. This means that the immune system was suppressed.

As shown in Fig. 4b, MPA, RAPA, and Cyc A from the nanofilms slightly decreased the activity of IL-2. There are only a few differences of released IL-2 from immune cell culture between no treatment and printed (PLL/HA/drugs) trilayer-based nanofilms. However, we can confirm remarkable differences of quantity of released IL-2 between the PLL/HA films and printed (PLL/HA/drugs) trilayer multilayered nanofilms, which means immunosuppressive drugs from printed films have clearly had a palpable effect. Additionally, under PHA stimulation, cell proliferation in the T lymphocyte culture (Fig. 4c) displays that IL-2 was obviously reduced by the effects of the immunosuppressive drugs released from the printed drugs nanofilms. The drug concentrations of ink solutions were 0.1 mg/mL MPA, 0.1 mg/mL Cyc A, and 20 ng/mL RAPA, indicating that the concentration of immunosuppressive drugs can be influential. Although the concentration of RAPA was small, its effects did not differ significantly compared to those of the other drugs.

**Figure 5:**
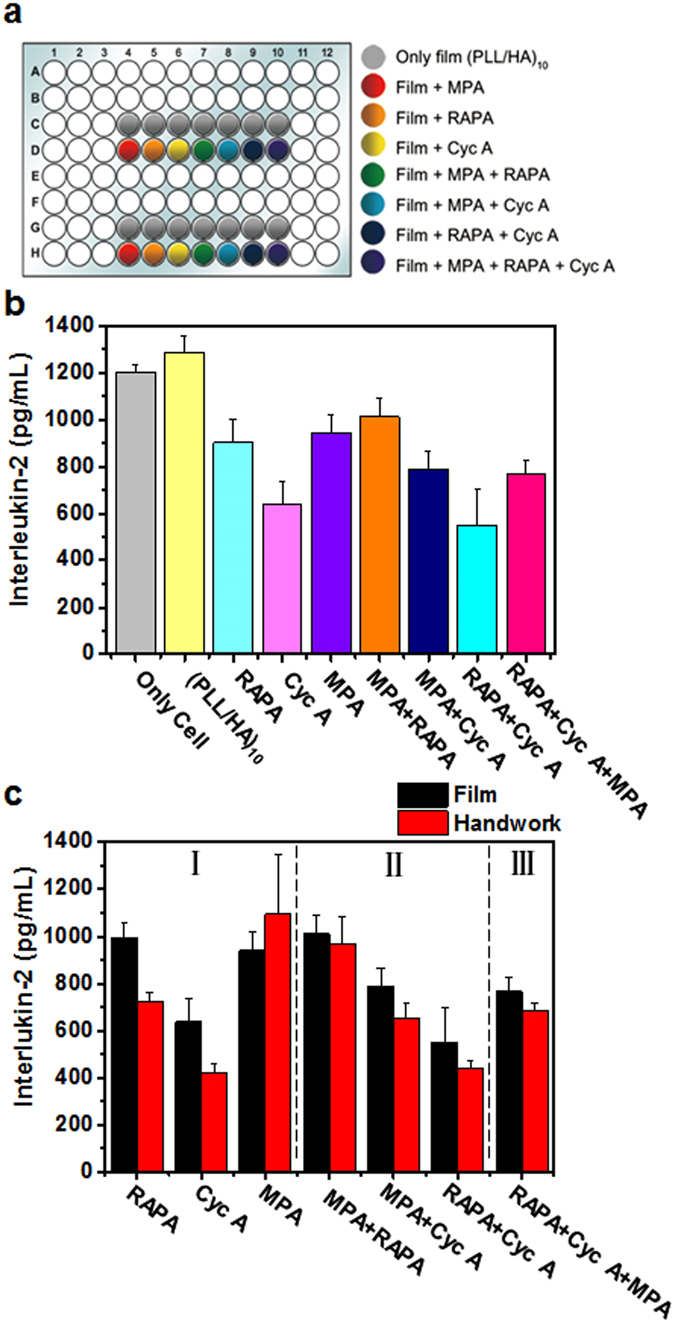
(**a**) Combinations of immunosuppressive drugs on the single plate (The figure was drawn by M.C). (**b**) Amounts of IL-2 released by interaction between immune T cells and immunosuppressive drugs. (**c**) IL-2 level comparison between the multilayer nanofilms and manual immunosuppressive drug combination test.

After we checked the immunosuppressive effect of each of the drugs in the printed nanofilms, we designed a diverse array of printed multilayer nanofilms that are made of various drug combinations on the single substrate (Fig. 5a). Using the diverse combinations array, obtaining enough data about interactions between immune T cell and immunosuppressive drugs in the film is capable in just one experiment. Figure 5b shows the amounts of released IL-2 from immune cell depending on the drug or drug combinations in the printed nanofilms. We confirmed the effect of printed nanofilms without drugs. In the case of PLL/HA printed nanofilms, IL-2 was released more than in the condition of only cells. Even though PLL and HA are generally known as nontoxic and biocompatible materials, sensitive immune T cells were slightly affected by film components. In nanofilms incorporated with only a single drug (MPA, RAPA, or Cyc A), the amount of released IL-2 depended on the specific drug in the printed nanofilms. In printed nanofilms incorporated with two drug combinations (MPA + RAPA, RAPA + Cyc A, or MPA + Cyc A), although amounts of immunosuppressive drugs increased, two-drug printer multilayer nanofilms did not show a more marked effect than single-drug nanofilms. In case of printed nanofilms with MPA, RAPA and Cyc A, amounts of IL-2 from immune T-cell is not always lower than amounts of IL-2 by printed nanofilms with single or two immunosuppressive drugs (Fig. 5c). More mixed immunosuppressive drugs are not necessarily good immunosuppressive effects. In addition, Fig. 4c shows that slight differences were observed with manual tests and when using the prepared kits. A greater amount of IL-2 was released by the films, compared to the manual tests. Interestingly, we could confirm immunosuppressive effects with only a single kit, as the amount of released IL-2 had various patterns depending on the specific drug combination. Therefore, it is important to determine the most suitable patient-specific drug combination.

Our results suggest that an inkjet-based high-throughput assembly system for fabricating a multi-component array can be effectively designed to develop a combination of desired immunosuppressive drugs on various substrates and to control the release pattern of the functional molecules for achieving suitable cell culture conditions. We prepared an immunosuppressive drug screening kit by printing small, constant, and uniform amounts of drugs. Biomaterial printing provides a functional application to the commercial inkjet printing technology, and could play an important role as an ink material that can be readily functionalized to extend printed forms. Tuning the structural properties of materials in water-based solutions with a co-solvent provides a common base material for applications in cell culture, tissue engineering, and biosensing. Furthermore, a large variety of bioinstructive molecules (e.g., growth factors, cytokines, and enzymes) can be stabilized within the printed material to provide an effective method to investigate cell–material interactions at the microscale.

## Methods

### Materials

Poly-L-lysine (PLL, MW = 1.5~3.0 k), mycophenolic acid (MPA), Cyclosporine A (Cyc A), rapamycin (RAPA), and phytohaemagglutinin (PHA) were purchased from Sigma-Aldrich. Dried Hyaluronic acid sodium (HA, MW = 10 k) was purchased from Lifecore Biomedical. Dimethyl sulfoxide (DMSO, 99.5%) was purchased from Daejung. The concentration of PLL (pH 8) and HA (pH 6) was 1 mg/mL in the co-solvent (DMSO: DI water 1:1 v/v). The concentration of MPA and Cyc A solutions were 0.1 mg/mL. The concentration of the RAPA solution was 20 ng/mL. PHA was dissolved in DMSO at 1 mg/mL. In general, DMSO plays an important role in cell culture, including use as a cryoprotectant for the frozen preservation of cultured cells, cell medium supplement to reduce ice formation, and a component of the polymerase chain reaction mix before reacting^38–40^.

### Ink-jet printing system

A commercial drop-on-demand piezoelectric inkjet photo printer (Epson R290) was used. It was equipped with a 6-nozzle line, with black, yellow, light magenta, magenta, light cyan, and cyan. The nozzle diameter was set at around 25 μm, with a droplet volume of 1.5–2 pL (Figure S1, see the Supporting Information for details).

### Fabrication of Polydimethylsiloxane(PDMS) packing

Using a SYLGARD 184 Silicone Elastomer Kit, the curing agent, at a 10:1 ratio, was mixed and poured into 96-well plate lid. The mixture was cured at 80 °C for 12 h. We then pierced the 96-well plate with shaped holes, in the prepared PDMS plates, using a 7 mm punching machine. The 96-well plate shaped PDMS plates were sterilized in autoclave at 121 °C for 15 min.

### Characterization of the printed nanofilms

The growth and thickness of the films were evaluated with a profilometer (Dektak 150). Field emission scanning electron microscopy (FE-SEM) images were obtained with Carl Zeiss instrument (model: SIGMA).

### T-cell activation. Dynabeads Human T-Activator CD3

28 Beads (Life Technologies) were used to activate T cells in the Peripheral blood mononuclear cell (PBMCs). After incubating (37 °C) PBMCs for 16 h (C.T.L. Ltd., U.S.A), PBMCs were placed on 96-well plates (2 × 105/well) containing 2 μL/well beads. We prepared beads and PBMCs at a 1:1 ratio. After eliminating supernatants and adding clean media, PHA solution was added to the well plates to stimulate the PBMCs. Media conditions were Roswell Park Memorial Institute medium (RPMI 1640), 10% fetal bovine serum (FBS), 5% CO2, and 1% penicillin streptomycin. (Figure S2, see the Supporting Information for details).

### ELISA assay

We measured the amount of interleukin-2 (IL-2) in aliquots separated from the cell cultured in a 96-well plate. IL-2 standard protein, monoclonal antibody, and biotin conjugated polyclonal antibody were purchased from Biolegend. Horseradish peroxidase (HRP) conjugated streptavidin and 2, 2′-Azino-bis(3-ethylbenzothiazoline-6-sulfonic acid)(ABTS) were purchased from Sigma. Briefly, 100 μL of the first antibody (monoclonal antibody dilution was 1:200) was coated on the ELISA plate at 4 °C, overnight 12 h. After washing with 2% Bovine serum albumin (BSA) buffer solution, the plate was cleaned with Phosphate Buffered Saline Tween-20 (PBST) and blocked for 2 h. After adding 100 μL of the polyclonal second antibody (1:2000 dilution) for 1 h, then 100 μL of HRP conjugated streptavidin (1:200 Dilution) for 30 min, and then adding 100 μL of ABTS for 15 min, the absorbance measurements were conducted at a wavelength of 410 nm. The amounts of TNF-alpha and released IL-2 were quantified with a standard graph.

## Electronic supplementary material

supporting information
