# Supplementary material for: Multicomponent High-throughput Drug Screening via Inkjet Printing to Verify the Effect of Immunosuppressive Drugs on Immune T Lymphocytes
Source: Sci Rep. 2017 Jul 24;7:6318. doi: 10.1038/s41598-017-06690-2 (PMC5524941; doi:10.1038/s41598-017-06690-2)
Supplement: Supplementary file 1 — supporting information [file 41598_2017_6690_MOESM1_ESM.doc]

**Multicomponent High-throughput Drug Screening via Inkjet Printing to Verify the Effect of Immunosuppressive Drugs on Immune T Lymphocytes**

**Moonhyun Choi^1^, Jangsun Hwang^2^, Jonghoon Choi^2,*^ and Jinkee Hong^1,*^**

^1^School of Chemical Engineering and Material Science, Chung-Ang University, 84 Heukseok-ro, Dongjak-gu, Seoul 06974, Republic of Korea

^2^School of Integrative Engineering, Chung-Ang University, Seoul 06974, Republic of Korea

^*^Corresponding author: E-mail address: jkhong@cau.ac.kr, Tel: +82-2-820-5561

Co-corresponding author: E-mail address: nanomed@cau.ac.kr, Tel: +82-2-820-5258


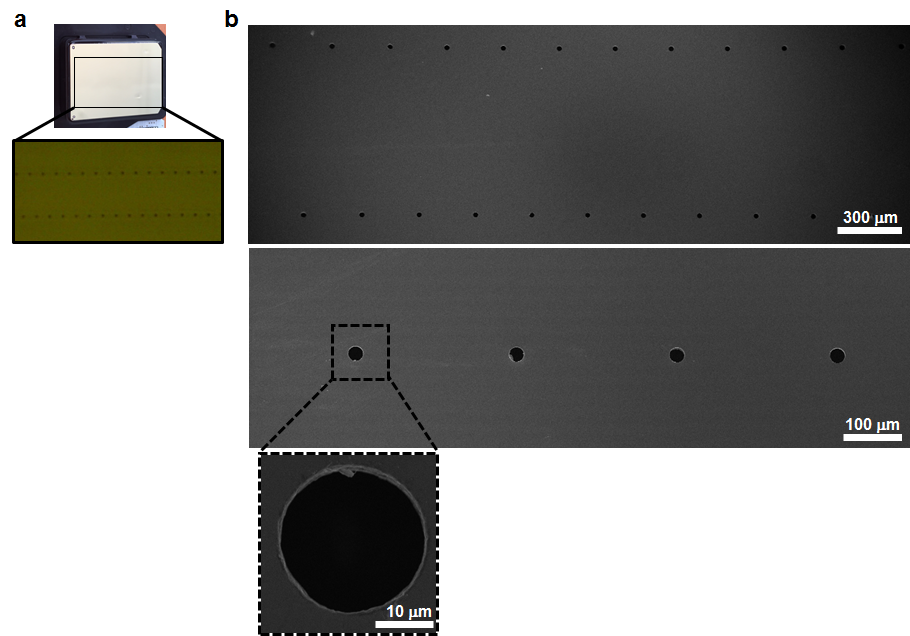


**Figure S1.** (a) Digital images of printer head. (b) FE-SEM images of nozzles in printer head.

**
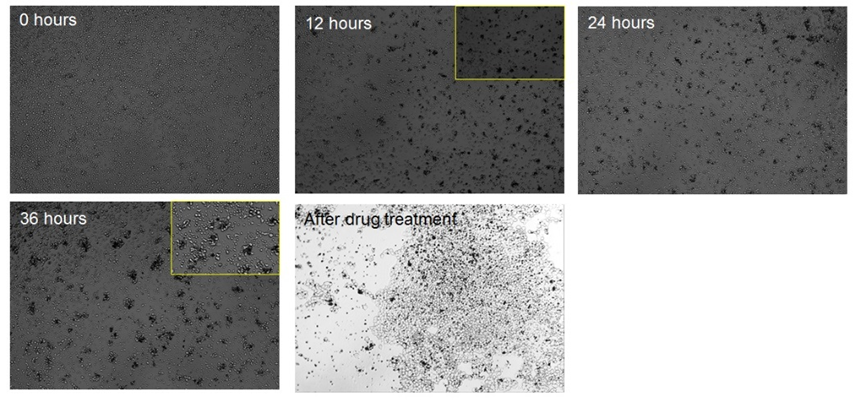
**

**Figure S2.** Microscopy images of activation of immune T cell over time (beads:cell = 1:1).


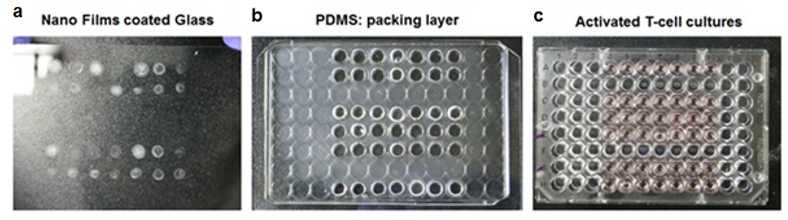


**Figure S3.** The digital images of (a) (PLL/HA/drugs) nano film-coated glass, (b) PDMS mold, and (c) well-plate for T cell culture.


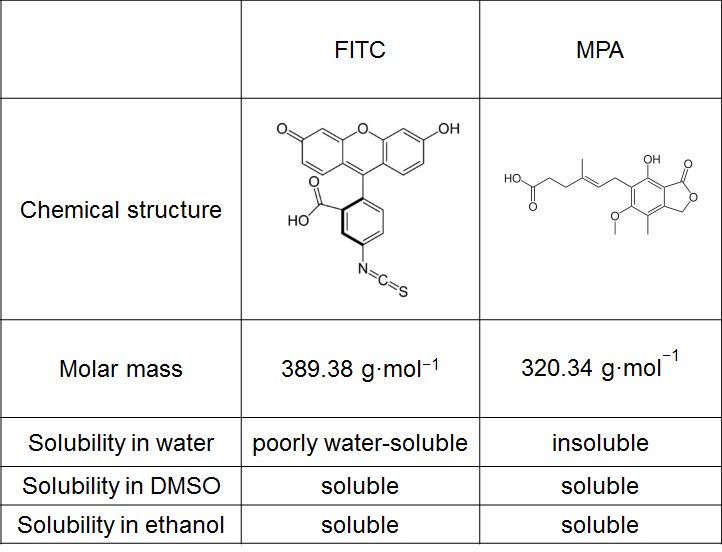


Table S1. Chemical structure, molar mass and solubility in solvent type of FITC and MPA.
